# Relationship of the Reported Intakes of Fat and Fatty Acids to Body Weight in US Adults

**DOI:** 10.3390/nu9050438

**Published:** 2017-04-28

**Authors:** Susan K Raatz, Zach Conrad, LuAnn K Johnson, Matthew J Picklo, Lisa Jahns

**Affiliations:** 1 Grand Forks Human Nutrition Research Center, USDA-ARS, Grand Forks, ND 58203, USA; zach.conrad@ars.usda.gov (Z.C.); luann.johnson@ars.usda.gov (L.KJ.); matthew.picklo@ars.usda.gov (M.JP.); lisa.jahns@ars.usda.gov (L.J.); 2 Food Science and Nutrition, University of Minnesota, Saint Paul, MN 55108, USA

**Keywords:** BMI, dietary intake, fat, fatty acids, saturated fat, monounsaturated fat, polyunsaturated fat, dietary intake, National Health and Nutrition Examination Survey (NHANES), What We Eat in America (WWEIA), J0101

## Abstract

Dietary fat composition may modulate energy expenditure and body weight. Little is known about the relationship between fatty acid intake and body weight at a population level. The purposes of this study were to compare intakes of energy, macronutrients, and individual fatty acids across BMI categories (1) for the US adult population and, (2) by sociodemographic groups. Reported dietary intake data from the National Health and Nutrition Examination Survey (NHANES) and What We Eat in America (WWEIA) surveys in the years 2005–2012 were analyzed. Overall, we found that the reported intake of carbohydrate, protein, total fat, total saturated fat (as well as long-chain saturated fatty acids 14:0–18:0), and monounsaturated fatty acids (MUFAs) were positively associated with BMI; while lauric acid (a medium-chain saturated fatty acid, 12:0) and total polyunsaturated fatty acids (PUFAs) (as well as all individual PUFAs) were not associated with BMI. Non-Hispanic black individuals demonstrated a negative association between BMI and energy intake and a positive association between total PUFAs, linoleic acid (LA), α-linolenic acid (ALA) and BMI. Individuals with less than a high school education showed a negative association between BMI and DHA. Mexican-Americans reported intakes with no association between BMI and energy, any macronutrient, or individual fatty acids. These findings support those of experimental studies demonstrating fatty acid-dependent associations between dietary fatty acid composition and body weight. Notably, we observed divergent results for some sociodemographic groups which warrant further investigation.

## 1. Introduction

Dietary fats and oils have traditionally been viewed solely for their energy content, on the basis that overconsumption leads to excess energy intake, the development of obesity and increased cardiometabolic disease risk. This view led to the promulgation of dietary recommendations based on total fat intake and, later, to broad recommendations about entire classes of fat (saturates vs. unsaturates). However, more recent evidence presents a more nuanced view; the metabolic and health effects of fats and oils may vary according to their specific fatty acid compositions and molecular structures [1]. Some fatty acids are consumed in relatively large quantities, such as linoleic (LA, 18:2n-6, 7% energy (%en), palmitic (16:0, 6%en) and oleic acids (18:1n-9, 11%en); however others, such as α-linolenic acid (ALA; 18:3n-3; 1%en) and the long-chain (LC) omega-3 (n-3) fatty acids, eicosapentaenoic (EPA, 20:5n-3; <0.02%en) and docosahexaenoic acids (DHA, 22:6n-3 < 0.3%en), are consumed in small quantities yet have potent biologic effects [2]. Under experimental conditions dietary fatty acids modulate energy balance due to the chain length, degree of unsaturation, and the position and configuration of double bonds which affect their metabolic fate [3,4]. Short- (C:2–4) and medium-chain (C:6–12) fatty acids increase energy expenditure due to their preferential oxidation vs. esterification, [5,6,7] but they are consumed in relatively low amounts (<1%en) in the American diet [2]. On the other hand, very-long-chain polyunsaturated fatty acids (PUFAs), such as long-chain (LC) n-3, undergo low amounts of β-oxidation [8], but increase total resting β-oxidation levels [9] and reduce body fat mass [10]. Randomized trials in overweight or obese humans demonstrate that energy restricted diets containing LCn-3 result in reduced body weight [11], or decreased body fat with no change in weight [12,13]. Conversely, studies in rats indicate that elevating the LA content of the diet increases the development of adiposity, potentially through elevation of endocannabinoid signaling [14].

Given these experimental data, we would expect heterogeneous associations between different fatty acids and obesity status among humans in observational studies. Yet, to the best of our knowledge these relationships have not been investigated in observational settings. We therefore aimed to examine the extent to which reported intake of selected fatty acids, from 24-h dietary recalls, were associated with the prevalence of obesity. Our objectives were to compare intakes of energy, macronutrients, and individual fatty acids across BMI categories for (1) the US adult population and, (2) sociodemographic subgroups.

## 2. Materials and Methods 

### 2.1. Survey Design and Data 

These analyses examined data from the 2005–2012 National Health and Nutrition Examination Survey (NHANES) and the What We Eat in America (WWEIA) survey, the dietary assessment component of NHANES. NHANES is a large, nationally representative continuous, cross-sectional survey of the health and nutrition behaviors of non-institutionalized civilian Americans. It includes a comprehensive medical examination and sociodemographic and health behavior questionnaires, along with dietary intake assessments administered by trained interviewers. Data are released in 2-year cycles and details may be found elsewhere [15].

### 2.2. Dietary Intake 

Trained interviewers conducted an in-person 24-h dietary recall using the USDA’s Automated Multiple-Pass Method [16]. A second dietary recall was administered by telephone 3–10 days later. In this analysis, day 1 intake data were used in accordance with the National Cancer Institute’s analytic recommendations because this provides a reliable estimate of mean intake at the population level [17]. Data were collected on the reported intake of energy, protein, carbohydrate, and fat as well as intake of saturated fatty acids (SFAs), monounsaturated fatty acids (MUFAs), and PUFAs. Specific fatty acids included were lauric acid (12:0), myristic acid (14:0), palmitic acid, stearic acid, oleic acid (18:1n-9), LA, ALA, and the LCn-3 fatty acids, EPA, docosapentaenoic acid (DPA; 22:5n-3) and DHA. 

### 2.3. Participants and Body Mass Index Categories

Adults 19 years and older who provided data on diet and sociodemographics were considered for this study. Females who reported being pregnant or lactating were excluded to reduce potential confounding due to their association with elevated BMI, and individuals who reported BMI < 18.5 (underweight) were excluded to reduce potential confounding due to their association with possible illness-related metabolic effects (*n* = 358). One individual reporting an unrealistic BMI of 130 kg/m^2^ was excluded, and individuals not providing data on educational attainment were also removed (*n* = 39). The final sample of this study was 19,916. Individuals were categorized as normal weight (BMI = 18.5–24.9 kg/m^2^), overweight (BMI = 25–29.9 kg/m^2^), obese—grade 1 (BMI = 30–34.9 kg/m^2^), obese—grade 2 (BMI = 35–39.9 kg/m^2^), and obese—grade 3 (BMI ≥ 40 kg/m^2^) [15]. 

### 2.4. Statistical Analysis

Demographic data included sex, age, race/ethnicity, and education level. Race and ethnicity were self-reported. The race/ethnic groups identified in the 2005–2012 survey cycles were non-Hispanic white, non-Hispanic black, Mexican-American, other Hispanic and other races, including multi-racial. Education level was categorized into three groups: less than high school diploma (<HS), high school diploma or equivalent (HS), and any post-secondary education (>HS). Beginning in 2007–2008, the NHANES data collection protocol changed to oversample some race/ethnicities to produce more reliable estimates for these groups. Therefore, to preserve consistency in how race/ethnicity data were collected across NHANES waves in this study, outcome data are reported just for non-Hispanic whites, non-Hispanic blacks, and Mexican-Americans, as recommended by NHANES analytic guidelines [18]. Statistical models were additionally adjusted for other Hispanics, multi-racial, and other race/ethnicities, which were combined into a single group (other race/ethnicities) because of small sample sizes.

The SURVEYREG procedure in SAS V9.4 (SAS Institute, Inc., Cary, NC, USA) was used to test for linear trends in nutrient intakes across the five BMI categories. Contrasts were included comparing the mean intake of the normal weight group to the other four BMI groups. The simulation method within the SURVEYREG procedure was used to adjust for multiple comparisons to the normal weight group. Education level and race/ethnicity were included as covariates in all analyses. 

All statistical analyses were adjusted for the NHANES survey design and the day 1 sample weights that are included with the dietary data. The sample weights account for differential probabilities of selection, nonresponse and noncoverage. Data are reported as percentages or means ± SE. All statistical tests were considered significant at *p* ≤ 0.05.

## 3. Results

### 3.1. Participant Characteristics

A total of 19,916 individuals were included in this analysis of dietary records obtained from the NHANES survey from 2005–2012. Table 1 illustrates the sex, race/ethnicity, educational and BMI categories of the population included in these analyses. The average age (mean ± SEM) of participants at screening by BMI category were: normal weight: 43.6 ± 0.6 years; overweight: 48.5 ± 0.4 years; obese—grade 1: 48.4 ± 0.3 years; obese—grade 2: 48.1 ± 0.5 years; and obese—grade 3: 46.6 ± 0.6 years. The normal weight subjects were significantly younger (*p* = 0.001) than the individuals in other weight categories. 

### 3.2. Relationship of BMI Category to Daily Intake of Energy, Macronutrients, and Individual Fatty Acids, Overall

Table 2 contains the reported intake of energy, macronutrients, and fatty acids of participants by BMI category. A significant negative relationship was observed in reported energy intake (mean ± SEM) by BMI category. Compared to the normal weight category, reported intake of carbohydrate was lower and that of both protein and total fat were higher in the overweight and all obese BMI categories.

The reported intake of total fat increased over BMI categories. This was associated with an increased intake of SFA and MUFA. No association was observed for lauric acid while increased intake over BMI categories was reported for the SFA, myristic acid, palmitic acid, stearic acid and the MUFA, oleic acid. No difference was observed in the reported intake of total PUFA, LA, or ALA between BMI classifications. Total LCn-3 intake was unchanged over BMI categories as was that of individual LCn-3. 

### 3.3. Relationship of BMI Category to Daily Intake of Energy, Macronutrients, and Individual Fatty Acids, by Sociodemographic Characteristics 

The reported intake of energy, macronutrients, and fatty acids of participants by BMI category and sex are presented below. Among men (Table 3), BMI was negatively associated with carbohydrate intake and positively associated with intake of protein, total fat, total SFA, myristic acid, palmitic acid, total MUFA, and oleic acid. We observed a trend for increased total PUFA and LA intake with increasing BMI among men but not women. Similar results were observed among women, except for a positive association between BMI and energy intake and no association with carbohydrate intake (Table 4).

Table 5 presents the reported intake of energy, macronutrients, and fatty acids of non-Hispanic Whites. BMI was negatively associated with carbohydrate intake and positively associated with intake of protein, total fat, total SFA, myristic acid and palmitic acids, and total MUFAs and oleic acid. No association was observed between PUFA intake and BMI. Non-Hispanic Blacks demonstrated a negative association between BMI and energy intake, and a positive association between total PUFA, LA, ALA and BMI (Table 6). No associations between BMI and any fatty acid were observed among Mexican-Americans (Table 7).

A negative association was observed between energy intake and DHA with BMI among individuals with less than high school education, and positive associations were observed between BMI and total fat, total SFA, palmitic acid, and stearic acid (Table 8). Among high school graduates, BMI was positively associated with total fat, total SFA, palmitic acid, total MUFA, oleic acid, total PUFA, and LA (Table 9). Among individuals with some post-secondary education, BMI was negatively associated with carbohydrate intake, and positively associated with total SFA, palmitic acid, total MUFA, and oleic acid; no associations were observed between BMI and PUFAs (Table 10).

## 4. Discussion

Experimental evidence indicates that selective fatty acids have physiologic impacts upon energy expenditure. In this work, we examined the extent to which the intake of different fatty acids, as derived from reported food intake from NHANES diet recalls (2005–2012), were associated with BMI status. Overall we found that the reported intake of carbohydrate was associated with a decreased BMI while protein, total fat, total saturated fat (as well as long-chain saturated fatty acids 14:0–18:0), and MUFAs were positively associated with BMI. Lauric acid (a medium-chain saturated fatty acid, 12:0) and total PUFAs (as well as all individual PUFAs) were not associated with BMI. Findings were consistent among most sociodemographic strata, making these results applicable to the vast majority of people in the population, with notable exceptions. Non-Hispanic blacks showed positive associations between BMI and total PUFA (including LA and ALA), individuals with less than a high school education showed a negative association between BMI and DHA, and Mexican-American individuals showed no associations between BMI and any macronutrient or individual fatty acids.

The relationship between fatty acid intake and obesity prevalence in humans from observational trials is inconsistent. These data relate high SFA intake to obesity and increased adiposity [19,20], particularly in obesity prone carriers of the fat-mass and obesity-associated (FTO) gene [21,22,23]. Our data concur with the relationship of SFA intake and increasing BMI, with the exception of the short-chain SFA, lauric acid. Inverse associations of adiposity have been noted with LCn-3 intake from fish [24]. We observed no relationship between LCn-3 and BMI as the intake of LCn-3 in our data is very low. We observed a positive relationship between MUFA and body weight but the literature reports inconsistent relationships for MUFA and BMI, visceral fat area and adipocyte size in both men and women [25].

Fatty acid saturation level affects the β-oxidation of fatty acids [9,26,27]. In humans, MUFA (particularly oleic acid) intake is associated with reduced deposition of adipose fat compared to isocaloric amounts of SFA [28,29], under conditions of both positive energy balance and energy restriction [29,30]. This is likely resulting from increased β-oxidation of fatty acids, and hence total energy expenditure (TEE) [27]. Experimental evidence shows that the consumption of PUFA is associated with reduced adiposity perhaps due to alterations in metabolism favoring fatty acid β-oxidation of lipid [31], elevated mitochondrial respiration in liver, cardiac and skeletal muscle cells [32] and increased expression of uncoupling protein-3 in skeletal muscle [33]. Micallef et al demonstrated that higher plasma levels of total n-3 fatty acids is associated with a reduction in BMI, waist circumference and hip circumference is a sample of adults with weights ranging from normal to obese [34].

The increased β-oxidation of unsaturated lipids is related to reductions in body fat mass. In a controlled feeding study, the intake of MUFAs with low SFAs resulted in reductions in abdominal fat mass [35]. PUFAs for SFA substitution in overweight and obese individuals in a 5-week crossover designed weight loss trial resulted in reduced abdominal fat mass with no difference in total weight loss [36]. Other studies show increased energy expenditure after both MUFA and PUFA compared to SFA intake in normal-weight individuals [37,38]. Our results showed that total MUFA and SFA, and oleic, myristic, palmitic and stearic acids intake were positively associated with BMI while PUFA and lauric acid were not. We were unable to assess neither whether the differences observed were due to energetics nor whether participants exhibit variation in regional fat distribution due to dietary fat sources. These questions warrant further investigation.

In our study we saw no association of reported total PUFA, ALA or LA intake to BMI category except among non-Hispanic blacks, which warrants further investigation. The intake of EPA and DHA was not associated with body weight even though both epidemiological [34,39] and experimental [10,13,40] evidence shows a positive link between increased intake of LCn-3 and reduced body weight. The reported intake of LCn-3 in this survey was very low in the total population with intake of EPA and DHA well below levels that have been shown to increase energy expenditure. Previous evaluations of LCn-3 intake from fish and other sources reveal low intake levels that are comparable to what we observed here [41,42].

We recognize that these data represent an analysis at the population level and cannot take into account individual responses to dietary fatty acid intake. Polymorphisms of varying frequencies and ethnic variability in numerous genes encoding proteins involved in various aspects of fatty acid metabolism are linked to the development of obesity [43,44]. These proteins include, but are not limited to fatty acid binding proteins (FABP), fatty acid amide hydrolase (FAAH), delta 6 desaturase (FADS2), the fatty acid transporter CD36, and peroxisomal proliferator activated receptor (PPAR) gamma [45,46,47,48,49]. The extent to which these polymorphisms modify energetic or satiety related responses of specific fatty acids requires further investigation. Recent data show that selective fatty acid intake in humans can alter DNA methylation and that selective fatty acids have epigenetic influences [50].

The total fat and fatty acid intake of our assessment of the NHANES diet records from 2005–2012 is substantially the same as that reported on the 1999–2000 NHANES survey when evaluating the mean responses for all age groups without adjustment for BMI [8]. However, some research suggests that fat intake, particularly from SFA and MUFA, is decreasing in the US [51]. If true, given our observations, this change may positively influence body weight trends in the future. However, the removal of trans-fat from the market will bring major changes in the food supply due to reformulation of many processed foods [52]. Production of food oils with increased MUFA to replace partially hydrogenated oils will modify dietary fatty acid intake in, as yet, unknown ways.

Limitations of this work are primarily due to those inherent in self-reported diet. Self-reported dietary intake data collected by 24-h recall suffers from both random and systematic error, particularly in energy intake. Some survey participants may not report their true intake in order to impress the survey administrators or simplify the survey process [53,54]. It is, however, considered the least biased method and the best for describing dietary intake at the population level [55]. Measurement error usually results in regression to the mean, making it difficult to detect associations, but in this research, we found strong relationships between dietary fatty acids and BMI categories. As we did not evaluate individual food consumption data, we are unable to comment on the role that food choices play in the observed differences in reported energy, macronutrient, and fatty acid intakes. In this observational study, we did not evaluate body composition relationships to fatty acid intake so we were unable to determine the effect of fatty acid intake on regional adiposity between groups.

Strengths of this study include the use of a large nationally representative sample of nearly 20,000 US adults, which was stratified by diverse sociodemographic characteristics in order to observe important differences between groups. Anthropometric measurements and dietary data were collected by trained investigators. Reported dietary intake data from four 2-year survey cycles were combined to provide statistically reliable estimates for the sex- and age-specific subgroups of interest.

## 5. Conclusions

In this study, we found that overall the reported intake of protein, total fat, total saturated fat (as well as long-chain saturated fatty acids 14:0–18:0), and MUFAs was positively associated with BMI; while carbohydrate was negatively associated and lauric acid (a medium-chain saturated fatty acid, 12:0) and total PUFAs (as well as all individual PUFAs) were not associated with BMI. These findings support those of experimental studies that demonstrate fatty acid-dependent associations between dietary fatty acid composition and body weight. Notably, we observed divergent results for some sociodemographic groups (e.g. non-Hispanic blacks), which warrant further investigation. Future changes in fatty acid intake, as a result of changing consumer food preferences or industry reformulation of fats and oils, may change the association of dietary fat and oil intake to BMI.

## Figures and Tables

**Table 1:** Subject characteristics, National Health and Nutrition Examination Survey (NHANES) 2005–2012 for 19,916 adults ≥ 19 years of age.

Variable	*n*	Weighted % of Sample	Mean ± SEM
Sex			
Male	10,090	49.4	
Female	9826	50.6	
Race/ethnicity			
Mexican-American	3392	8.2	
Non-Hispanic black	4397	11.5	
Non-Hispanic white	9063	69.4	
Other Hispanic Other/multi–racial Education	1777 1387	4.9 6.1	
<HS	5466	17.8	
HS	4678	23.5	
HS	9772	58.7	
BMI category			BMI (kg/m^2^)
Normal weight	5785	31.0	22.4 ± 0.03
Overweight	6758	33.8	27.4 ± 0.03
Obese—grade 1	4186	20.3	32.2 ± 0.03
Obese—grade 2	1855	8.6	37.2 ± 0.05
Obese—grade 3	1332	6.3	45.4 ± 0.20

HS, high school diploma or equivalent.

**Table 2:** Dietary intake of energy, macronutrients, and fatty acids by BMI category: United States, NHANES 2005–2012 for adults ≥ 19 years of age ^1^.

Nutrient	Normal Weight *n* = 5785	Overweight *n* = 6758	Obese—Grade 1 *n* = 4186	Obese—Grade 2 *n* = 1855	Obese—Grade 3 *n* = 1332	*p* for Linear Trend
Energy (kcal)	2174 ± 23	2181 ± 16	2121 ± 24	2117 ± 26	2108 ± 36	0.02
Carbohydrate (g)	262 ± 1	257 ± 1 *	254 ± 1 *	254 ± 2 *	256 ± 2 *	0.004
Protein (g)	80 ± 1	82 ± 1 *	83 ± 1 *	82 ± 1 *	84 ± 1 *	0.002
Fat, total (g)	77 ± 1	79 ± 1 *	80 ± 1 *	83 ± 1 *	83 ± 1 *	<0.0001
SFA, total (g)	25.1 ± 0.2	25.6 ± 0.1	26.1 ± 0.2 *	27.2 ± 0.3 *	27.6 ± 0.3 *	<0.0001
Lauric acid (12:0) (g)	0.7 ± 0.0	0.7 ± 0.0	0.7 ± 0.0	0.8 ± 0.1	0.7 ± 0.1	0.60
Myristic acid (14:0) (g)	2.1 ± 0.1	2.1 ± 0.1	2.1 ± 0.1	2.2 ± 0.1	2.3 ± 0.1 *	0.0002
Palmitic acid (16:0) (g)	13.6 ± 0.1	13.9 ± 0.1	14.2 ± 0.1 *	14.7 ± 0.2 *	15.0 ± 0.2 *	<0.0001
Stearic acid (18:0) (g)	6.3 ± 0.1	6.6 ± 0.1 *	6.8 ± 0.1 *	7.0 ± 0.1 *	7.1 ± 0.1 *	<0.0001
MUFA, total (g)	28.1 ± 0.2	28.7 ± 0.2 *	29.4 ± 0.2 *	30.1 ± 0.3 *	30.0 ± 0.3 *	<0.0001
Oleic acid (18:1n-9) (g)	26.2 ± 0.2	26.8 ± 0.2 *	27.5 ± 0.2*	28.1 ± 0.3 *	28.0 ± 0.3 *	<0.0001
PUFA, total (g)	17.1 ± 0.2	17.7 ± 0.2	17.9 ± 0.2 *	18.0 ± 0.3 *	17.9 ± 0.4	0.06
LA (18:2n-6) (g)	15.1 ± 0.2	15.6 ± 0.2	15.9 ± 0.2 *	15.9 ± 0.2 *	15.8 ± 0.3	0.06
ALA (18:3n-3) (g)	1.5 ± 0.0	1.6 ± 0.0	1.6 ± 0.0	1.6 ± 0.0	1.6 ± 0.0	0.36
EPA (20:5n-3 (g)	0.04 ± 0.00	0.04 ± 0.00	0.03 ± 0.00	0.04 ± 0.01	0.03 ± 0.01	0.43
DPA (22:5n-3) (g)	0.02 ± 0.00	0.02 ± 0.00	0.02 ± 0.00	0.02 ± 0.00	0.02 ± 0.00	0.37
DHA (22:6n-3) (g)	0.08 ± 0.01	0.08 ± 0.01	0.07 ± 0.00	0.08 ± 0.01	0.07 ± 0.01	0.38
LCn-3, total (g)	0.14 ± 0.01	0.14 ± 0.01	0.13 ± 0.01	0.14 ± 0.01	0.13 ± 0.01	0.50

^1^ Data are presented as mean ± SEM; * *p* < 0.05 compared to the normal weight category. SFA, saturated fatty acid; MUFA, monounsaturated fatty acid, PUFA, polyunsaturated fatty acid; LA, linoleic acid; ALA, alpha linolenic acid; EPA, eicosapentaenoic acid; DPA, docosapentaenoic acid; DHA, docosahexaenoic acid; LCn-3, long chain omega-3.

**Table 3:** Dietary intake of energy, macronutrients, and fatty acids by BMI category: United States, NHANES 2005–2012 for Men ≥ 19 years of age (*n* = 10,090) ^1^.

Nutrient	Normal Weight *n* = 2834	Overweight *n* = 3899	Obese—Grade 1 *n* = 2147	Obese—Grade 2 *n* = 759	Obese—Grade 3 *n* = 451	*p* for Linear Trend
Energy (kcal)	2653 ± 35	2505 ± 23 *	2461 ± 34 *	2511 ± 37 *	2576 ± 63	0.29
Carbohydrate (g)	302 ± 2	293 ± 2 *	288 ± 2 *	287 ± 3 *	285 ± 5 *	<0.01
Protein (g)	94 ± 1	97 ± 1 *	97 ± 1	99 ± 2 *	100 ± 2 *	<0. 01
Fat, total (g)	89 ± 1	92 ± 1	94 ± 1 *	96 ± 1 *	98 ± 2 *	<0.0001
SFA, total (g)	28.8 ± 0.4	30.0 ± 0.2 *	30.5 ± 0.3 *	31.4 ± 0.5 *	32.3 ± 06 *	<0.0001
Lauric Acid (12:0) (g)	0.8 ± 0.0	0.8 ± 0.0	0.7 ± 0.0	0.7 ± 0.1	0.8 ± 0.1	0.59
Myristic Acid (14:0) (g)	2.3 ± 0.0	2.5 ± 0.0	2.4 ± 0.1	2.5 ± 0.1	2.6 ± 0.1 *	0.01
Palmitic Acid (16:0) (g)	15.8 ± 0.2	16.3 ± 0.1	16.7 ± 0.2 *	17.3 ± 0.3 *	17.7 ± 0.4 *	<0.0001
Stearic Acid (18:0) (g)	7.3 ± 0.1	7.7 ± 0.1 *	8.0 ± 0.1 *	8.2 ± 0.2 *	8.4 ± 0.2 *	<0.0001
MUFA, total (g)	32.8 ± 0.3	33.6 ± 0.2	34.7 ± 0.3 *	35.5 ± 0.6 *	36.3 ± 0.8 *	<0.0001
Oleic Acid (18:1n-9) (g)	30.6 ± 0.3	31.3 ± 0.2	32.4 ± 0.3 *	33.0 ± 0.5 *	33.8 ± 0.8 *	<0.0001
PUFA, total (g)	19.4 ± 0.3	19.9 ± 0.3	20.5 ± 0.3	20.7 ± 0.5	20.9 ± 0.9	0.07
LA (18:2n-6) (g)	17.2 ± 0.3	17.5 ± 0.3	18.2 ± 0.3	18.3 ± 0. 5	18.5 ± 0.8	0.06
ALA (18:3n-3) (g)	1.7 ± 0.0	1.8 ± 0.0	1.7 ± 0.0	1.8 ± 0.1	1.8 ± 0.1	0.14
EPA (20:5n-3 (g)	0.05 ± 0.00	0.05 ± 0.00	0.04 ± 0.00	0.05 ± 0.01	0.04 ± 0.01	0.43
DPA (22:5n-3) (g)	0.02 ± 0.00	0.03 ± 0.00	0.03 ± 0.00	0.03 ± 0.00	0.03 ± 0.00	0.35
DHA (22:6n-3) (g)	0.09 ± 0.01	0.09 ± 0.00	0.09 ± 0.01	0.09 ± 0.01	0.08 ± 0.01	0.50
LCn-3, total (g)	0.16 ± 0.01	0.16 ± 0.01	0.16 ± 0.01	0.16 ± 0.02	0.14 ± 0.02	0.50

^1^ Data are presented as mean ± SEM; * *p* < 0.05 compared to the normal weight category. SFA, saturated fatty acid; MUFA, monounsaturated fatty acid, PUFA, polyunsaturated fatty acid; LA, linoleic acid; ALA, alpha linolenic acid; EPA, eicosapentaenoic acid; DPA, docosapentaenoic acid; DHA, docosahexaenoic acid; LCn-3, long chain omega-3.

**Table 4:** Dietary intake of energy, macronutrients, and fatty acids by BMI category: United States, NHANES 2005–2012 for Women ≥ 19 years of age (*n* = 9826) ^1^.

Nutrient	Normal Weight *n* = 2951	Overweight *n* = 2859	Obese—Grade 1 *n* = 2039	Obese—Grade 2 *n* = 1096	Obese—Grade 3 *n* = 881	*p* for Linear Trend
Energy (kcal)	1804 ± 17	1741 ± 18 *	1724 ± 18 *	1829 ± 26	1863 ± 34	0.02
Carbohydrate (g)	221 ± 1	220 ± 1	219 ± 2	218 ± 2	221 ± 2	0.73
Protein (g)	66 ± 1	66 ± 1	68 ± 1	67 ± 1	69 ± 1	0.02
Fat, total (g)	64 ± 0	66 ± 0 *	67 ± 1 *	68 ± 1 *	68 ± 1 *	<0.001
SFA, total (g)	21.0 ± 0.2	21.2 ± 0.2	21.8 ± 0.3	22.7 ± 0.4 *	22.6 ± 0.4 *	<0.0001
Lauric acid (12:0) (g)	0.6 ± 0.0	0.7 ± 0.0	0.7 ± 0.0	0.7 ± 0.0	0.6 ± 0.0	0.54
Myristic acid (14:0) (g)	1.8 ± 0.0	1.8 ± 0.0	1.8 ± 0.0	1.9 ± 0.1	1.9 ± 0.1	0.03
Palmitic acid (16:0) (g)	11.3 ± 0.1	11.4 ± 0.1	11.7 ± 0.1 *	12.1 ± 0.2 *	12.2 ± 0.2 *	<0.0001
Stearic acid (18:0) (g)	5.3 ± 0.1	5.4 ± 0.1	5.5 ± 0.1 *	5.8 ± 0.1 *	5.8 ± 0.1 *	<0.0001
MUFA, total (g)	23.2 ± 0.2	23.8 ± 0.2	24.0 ± 0.3	24.7 ± 0.3 *	24.1 ± 0.3 *	<0.01
Oleic acid (18:1n-9) (g)	21.7 ± 0.2	22.3 ± 0.2	22.4 ± 0.3	23.0 ± 0.3 *	22.5 ± 0.3	<0.01
PUFA, total (g)	14.7 ± 0.2	15.6 ± 0.2 *	15.3 ± 0.2	15.2 ± 0.3	14.8 ± 0.3	0.73
LA (18:2n-6) (g)	13.0 ± 0.2	13.8 ± 0.2 *	13.5 ± 0.2	13.4 ± 0.3	13.0 ± 0.3	0.76
ALA (18:3n-3) (g)	1.3 ± 0.0	1.4 ± 0.0 *	1.4 ± 0.0	1.3 ± 0.0	1.3 ± 0.0	0.18
EPA (20:5n-3 (g)	0.04 ± 0.00	0.03 ± 0.00	0.03 ± 0.00	0.04 ± 0.01	0.03 ± 0.01	0.61
DPA (22:5n-3) (g)	0.02 ± 0.00	0.02 ± 0.00	0.02 ± 0.00	0.02 ± 0.00	0.02 ± 0.00	0.50
DHA (22:6n-3) (g)	0.07 ± 0.01	0.06 ± 0.0	0.06 ± 0.00	0.07 ± 0.01	0.06 ± 0.01	0.57
LCn-3, total (g)	0.12 ± 0.01	0.11 ± 0.01	0.11 ± 0.01	0.12 ± 0.02	0.11 ± 0.02	0.67

^1^ Data are presented as mean ± SEM; * *p* < 0.05 compared to the normal weight category. SFA, saturated fatty acid; MUFA, monounsaturated fatty acid, PUFA, polyunsaturated fatty acid; LA, linoleic acid; ALA, alpha linolenic acid; EPA, eicosapentaenoic acid; DPA, docosapentaenoic acid; DHA, docosahexaenoic acid; LCn-3, long chain omega-3.

**Table 5:** Dietary intake of energy, macronutrients, and fatty acids by BMI category: United States, NHANES 2005–2012 for Non-Hispanic Whites ≥ 19 years of age (*n* = 9063) ^1^.

Nutrient	Normal Weight *n* = 2827	Overweight *n* = 3123	Obese—Grade 1 *n* = 1788	Obese—Grade 2 *n* = 801	Obese—Grade 3 *n* = 524	*p* for Linear Trend
Energy (kcal)	2195 ± 29	2213 ± 21	2159 ± 32	2142 ± 34	2182 ± 48	0.35
Carbohydrate (g)	264 ± 2	257 ± 1 *	252 ± 2 *	254 ± 3 *	256 ± 4	0.015
Protein (g)	80 ± 1	83 ± 1 *	84 ± 1 *	84 ± 1 *	86 ± 2 *	0.005
Fat, total (g)	80 ± 1	82 ± 1 *	84 ± 1 *	87 ± 1 *	87 ± 1 *	<0.0001
SFA, total (g)	26.6 ± 0.3	27.3 ± 0.2	28.0 ± 0.3 *	29.1 ± 0.4 *	29.7 ± 0.5 *	<0.0001
Lauric acid (12:0) (g)	0.8 ± 0.0	0.8 ± 0.0	0.8 ± 0.0	0.9 ± 0.0	0.8 ± 0.1	0.88
Myristic acid (14:0) (g)	2.3 ± 0.04	2.4 ± 0.03	2.4 ± 0.04	2.5 ± 0.06	2.6 ± 0.08 *	<0.01
Palmitic acid (16:0) (g)	14.2 ± 0.2	14.5 ± 0.1	15.0 ± 0.2 *	15.6 ± 0.2 *	15.9 ± 0.2 *	<0.0001
Stearic acid (18:0) (g)	6.7 ± 0.1	7.0 ± 0.1 *	7.2 ± 0.1 *	7.5 ± 0.1 *	7.6 ± 0.1 *	<0.0001
MUFA, total (g)	28.9 ± 0.2	29.7 ± 0.2 *	30.6 ± 0.3 *	31.3 ± 0.4 *	31.4 ± 0.5 *	<0.0001
Oleic acid (18:1n-9) (g)	27.0 ± 0.2	27.8 ± 0.2 *	28.6 ± 0.3 *	29.2 ± 0.4 *	29.3 ± 0.5 *	<0.0001
PUFA, total (g)	17.5 ± 0.2	18.2 ± 0.2	18.4 ± 0.3	18.7 ± 0.4 *	18.4 ± 0.6	0.10
LA (18:2n-6) (g)	15.5 ± 0.2	16.0 ± 0.2	16.3 ± 0.3	16.5 ± 0.3 *	16.3 ± 0.5	0.10
ALA (18:3n-3) (g)	1.6 ± 0.0	1.7 ± 0.0	1.6 ± 0.0	1.7 ± 0.0	1.6 ± 0.15	0.40
EPA (20:5n-3 (g)	0.04 ± 0.00	0.04 ± 0.00	0.03 ± 0.00	0.04 ± 0.01	0.03 ± 0.01	0.73
DPA (22:5n-3) (g)	0.02 ± 0.00	0.02 ± 0.00	0.02 ± 0.00	0.02 ± 0.00	0.02 ± 0.00	0.13
DHA (22:6n-3) (g)	0.07 ± 0.01	0.07 ± 0.00	0.06 ± 0.01	0.08 ± 0.01	0.06 ± 0.01	0.80
LCn-3, total (g)	0.12 ± 0.01	0.12 ± 0.01	0.12 ± 0.01	0.14 ± 0.02	0.12 ± 0.02	0.92

^1^ Data are presented as mean ± SEM; * *p* < 0.05 compared to the Normal Weight category. SFA, saturated fatty acid; MUFA, monounsaturated fatty acid, PUFA, polyunsaturated fatty acid; LA, linoleic acid; ALA, alpha linolenic acid; EPA, eicosapentaenoic acid; DPA, docosapentaenoic acid; DHA, docosahexaenoic acid; LCn-3, long chain omega-3.

**Table 6:** Dietary intake of energy, macronutrients, and fatty acids by BMI category: United States, NHANES 2005–2012 for Non-Hispanic Blacks ≥ 19 years of age (*n* = 4397) ^1^.

Nutrient	Normal Weight *n* = 1075	Overweight *n* = 1284	Obese—Grade 1 *n* = 1027	Obese—Grade 2 *n* = 514	Obese—Grade 3 *n* = 497	*p* for Linear Trend
Energy (kcal)	2316 ± 50	2161 ± 42 *	2062 ± 40 *	2072 ± 60 *	1941 ± 40 *	<0.0001
Carbohydrate (g)	256 ± 3	254 ± 2	256 ± 3	256 ± 3	254 ± 3	0.82
Protein (g)	77 ± 1	80 ± 1	78 ± 1	78 ± 1	81 ± 2	0.22
Fat, total (g)	79 ± 1	81 ± 1	80 ± 1	82 ± 1	82 ± 1	0.03
SFA, total (g)	25.4 ± 0.4	25.5 ± 0.4	25.3 ± 0.3	25.7 ± 0.4	25.9 ± 0.4	0.28
Lauric acid (12:0) (g)	0.7 ± 0.0	0.7 ± 0.1	0.7 ± 0.0	0.7 ± 0.1	0.7 ± 0.1	0.48
Myristic acid (14:0) (g)	1.9 ± 0.1	1.9 ± 0.1	1.9 ± 0.1	1.9 ± 0.1	2.0 ± 0.1	0.76
Palmitic acid (16:0) (g)	14.1 ± 0.2	14.1 ± 0.2	14.0 ± 0.2	14.2 ± 0.2	14.4 ± 0.2	0.35
Stearic acid (18:0) (g)	6.6 ± 0.1	6.7 ± 0.1	6.6 ± 0.1	6.8 ± 0.1	6.7 ± 0.1	0.20
MUFA, total (g)	29.2 ± 0.4	29.7 ± 0.3	29.5 ± 0.4	30.0 ± 0.4	29.8 ± 0.3	0.17
Oleic acid (18:1n-9) (g)	27.2 ± 0.4	27.7 ± 0.3	27.6 ± 0.3	28.0 ± 0.4	27.8 ± 0.3	0.20
PUFA, total (g)	17.7 ± 0.3	18.4 ± 0.3	18.4 ± 0.3	18.6 ± 0.4	18.9 ± 0.5	0.01
LA (18:2n-6) (g)	15.7 ± 0.3	16.3 ± 0.3	16.3 ± 0.2	16.5 ± 0.4	16.7 ± 0.5	0.01
ALA (18:3n-3) (g)	1.4 ± 0.0	1.5 ± 0.0	1.5 ± 0.0	1.5 ± 0.0	1.6 ± 0.1	<0.01
EPA (20:5n-3 (g)	0.04 ± 0.00	0.04 ± 0.00	0.04 ± 0.01	0.03 ± 0.00	0.05 ± 0.01	0.44
DPA (22:5n-3) (g)	0.02 ± 0.00	0.03 ± 0.00	0.03 ± 0.00	0.03 ± 0.00	0.03 ± 0.00	0.32
DHA (22:6n-3) (g)	0.08 ± 0.01	0.08 ± 0.01	0.09 ± 0.01	0.07 ± 0.01	0.09 ± 0.01	0.49
LCn-3, total (g)	0.14 ± 0.01	0.15 ± 0.01	0.15 ± 0.07	0.13 ± 0.01	0.17 ± 0.02	0.44

^1^ Data are presented as mean ± SEM; * *p* < 0.05 compared to the Normal Weight category. SFA, saturated fatty acid; MUFA, monounsaturated fatty acid, PUFA, polyunsaturated fatty acid; LA, linoleic acid; ALA, alpha linolenic acid; EPA, eicosapentaenoic acid; DPA, docosapentaenoic acid; DHA, docosahexaenoic acid; LCn-3, long chain omega-3.

**Table 7:** Dietary intake of energy, macronutrients, and fatty acids by BMI category: United States, NHANES 2005–2012 for Mexican-Americans ≥ 19 years of age (*n* = 3292).

	Normal Weight *n* = 723	Overweight *n* = 1282	Obese—Grade 1 *n* = 795	Obese—Grade 2 *n* = 314	Obese—Grade 3 *n* = 178	*p* for Linear Trend
Energy (kcal) Carbohydrate (g)	2255 ± 41 272 ± 3	2206 ± 43 268 ± 3	2167 ± 37 272 ± 3	2082 ± 74 264 ± 4	2181 ± 85 265 ± 10	0.18 0.42
Protein (g)	83 ± 1	88 ± 1	85 ± 1	87 ± 2	83 ± 3	0.82
Fat, total (g)	77 ± 1	76 ± 1	76 ± 1	79 ± 1	79 ± 2	0.16
SFA, total (g)	25.1 ± 0.5	24.3 ± 0.4	24.2 ± 0.4	26.0 ± 0.6	26.1 ± 1.0	0.12
Lauric acid (12:0) (g)	0.6 ± 0.1	0.6 ± 0.1	0.6 ± 0.0	0.6 ± 0.0	0.7 ± 0.1	0.30
Myristic acid (14:0) (g)	2.0 ± 0.1	1.9 ± 0.1	2.0 ± 0.1	2.1 ± 0.1	2.1 ± 0.2	0.21
Palmitic acid (16:0) (g)	13.9 ± 0.2	13.6 ± 0.2	13.4 ± 0.2	14.3 ± 0.3	14.3 ± 0.5	0.15
Stearic acid (18:0) (g)	6.4 ± 0.2	6.2 ± 0.1	6.1 ± 0.1	6.7 ± 0.2	6.6 ± 0.3	0.19
MUFA, total (g)	28.6 ± 0.5	28.0 ± 0.4	27.8 ± 0.4	29.5 ± 0.6	29.2 ± 0.9	0.23
Oleic acid (18:1n-9) (g)	26.7 ± 0.5	26.1 ± 0.4	25.9 ± 0.4	27.5 ± 0. 6	27.3 ± 0.9	0.23
PUFA, total (g)	16.6 ± 0.3	16.4 ± 0.3	17.1 ± 0.3	16.4 ± 0.6	16.8 ± 0.6	0.84
LA (18:2n-6) (g)	14.7 ± 0.3	14.5 ± 0.3	15.1 ± 0.3	14.5 ± 0.5	14.9 ± 0.5	0.79
ALA (18:3n-3) (g)	1.5 ± 0.0	1.4 ± 0.0	1.5 ± 0.0	1.4 ± 0.0	1.4 ± 0.1	0.46
EPA (20:5n-3 (g)	0.04 ± 0.01	0.04 ± 0.01	0.03 ± 0.01	0.03 ± 0.01	0.04 ± 0.01	0.59
DPA (22:5n-3) (g)	0.02 ± 0.00	0.02 ± 0.00	0.03 ± 0.00	0.02 ± 0.00	0.02 ± 0.00	0.85
DHA (22:6n-3) (g)	0.08 ± 0.02	0.08 ± 0.01	0.08 ± 0.01	0.07 ± 0.01	0.08 ± 0.01	0.62
LCn-3, total (g)	0.14 ± 0.03	0.15 ± 0.01	0.13 ± 0.02	0.12 ± 0.02	0.14 ± 0.03	0.62

SFA, saturated fatty acid; MUFA, monounsaturated fatty acid, PUFA, polyunsaturated fatty acid; LA, linoleic acid; ALA, alpha linolenic acid; EPA, eicosapentaenoic acid; DPA, docosapentaenoic acid; DHA, docosahexaenoic acid; LCn-3, long chain omega-3.

**Table 8:** Dietary intake of energy, macronutrients, and fatty acids by BMI category: United States, NHANES 2005–2012 for adults ≥ 19 years of age with <High School education (*n* = 5466) ^1^.

Nutrient	Normal Weight *n* = 1403	Overweight *n* = 1946	Obese—Grade 1 *n* = 1217	Obese—Grade 2 *n* = 538	Obese—Grade 3 *n* = 362	*p* for Linear Trend
Energy (kcal)	2206 ± 44	2086 ± 38	2009 ± 55 *	1981 ± 53 *	1956 ± 84 *	0.004
Carbohydrate (g)	250 ± 3	249 ± 2	248 ± 3	240 ± 4	251 ± 4	0.55
Protein (g)	76 ± 1	78 ± 1	78 ± 1	80 ± 2	77 ± 2	0.19
Fat, total (g)	71 ± 1	72 ± 1	72 ± 1	75 ± 1 *	75 ± 2	0.03
SFA, total (g)	23.3 ± 0.4	23.4 ± 0.3	23.4 ± 0.4	25.1 ± 0.6 *	24.3 ± 0.6	0.02
Lauric acid (12:0) (g)	0.7 ± 0.5	0.7 ± 0.03	0.7 ± 0.05	0.6 ± 0.05	0.6 ± 0.04	0.49
Myristic acid (14:0) (g)	1.9 ± 0.1	1.9 ± 0.1	1.9 ± 0.1	2.1 ± 0.1	2.0 ± 0.1	0.12
Palmitic acid (16:0) (g)	12.8 ± 0.2	12.9 ± 0.2	12.8 ± 0.3	13.8 ± 0.3	13.4 ± 0.3	0.01
Stearic acid (18:0) (g)	6.0 ± 0.1	6.1 ± 0.1	6.1 ± 0.1	6.5 ± 0.1 *	6.2 ± 0.2	0.04
MUFA, total (g)	26.2 ± 0.4	26.3 ± 0.3	26.4 ± 0.4	27.5 ± 0.5	27.3 ± 0.7	0.06
Oleic acid (18:1n-9) (g)	24.4 ± 0.3	24.6 ± 0.3	24.6 ± 0.4	25.6 ± 0.5	25.4 ± 0.7	0.08
PUFA, total (g)	15.2 ± 0.3	15.7 ± 0.4	16.0 ± 0.3	15.6 ± 0.4	16. 6 ± 0.9	0.19
LA (18:2n-6) (g)	13.4 ± 0.3	13.7 ± 0.3	14.1 ± 0.3	13.8 ± 0.3	14.7 ± 0.9	0.20
ALA (18:3n-3) (g)	1.3 ± 0.0	1.4 ± 0.1	1.4 ± 0.0	1. 4 ± 0.0	1.5 ± 0.1	0.17
EPA (20:5n-3 (g)	0.04 ± 0.01	0.03 ± 0.03	0.04 ± 0.04	0.04 ± 0.01	0.03 ± 0.01	0.13
DPA (22:5n-3) (g)	0.02 ± 0.00	0.02 ± 0.00	0.02 ± 0.00	0.02 ± 0.00	0.02 ± 0.00	0.47
DHA (22:6n-3) (g)	0.08 ± 0.01	0.06 ± 0.00	0.07 ± 0.01	0.07 ± 0.01	0.05 ± 0.01 *	0.04
LCn-3, total (g)	0.14 ± 0.02	0.11 ± 0.01	0.13 ± 0.01	0.12 ± 0.02	0.10 ± 0.01	0.07

^1^ Data are presented as mean ± SEM; * *p* < 0.05 compared to the Normal Weight category. SFA, saturated fatty acid; MUFA, monounsaturated fatty acid, PUFA, polyunsaturated fatty acid; LA, linoleic acid; ALA, alpha linolenic acid; EPA, eicosapentaenoic acid; DPA, docosapentaenoic acid; DHA, docosahexaenoic acid; LCn-3, long chain omega-3.

**Table 9:** Dietary intake of energy, macronutrients, and fatty acids by BMI category: United States, NHANES 2005–2012 for adults ≥ 19 years of age with High School education (*n* = 4678) ^1^.

Nutrient	Normal Weight *n* = 1297	Overweight *n* = 1546	Obese—Grade 1 *n* = 1011	Obese—Grade 2 *n* = 488	Obese—Grade 3 *n* = 336	*p* for Linear Trend
Energy (kcal)	2187 ± 41	2228 ± 41	2166 ± 50	2080 ± 53	2219 ± 57	0.53
Carbohydrate (g)	266 ± 3	264 ± 2	261 ± 3	262 ± 3	260 ± 4	0.27
Protein (g)	80 ± 1	81 ± 1	83 ± 1	84 ± 1	83 ± 2	0.06
Fat, total (g)	80 ± 1	80 ± 1	82 ± 1	84 ± 1 *	87 ± 2 *	<0.0001
SFA, total (g)	26.5 ± 0.4	25.9 ± 0.4	27.0 ± 0.4	28.0 ± 0.5	28.6 ± 0.7 *	<0.001
Lauric acid (12:0) (g)	0.8 ± 0.0	0.7 ± 0.0	0.8 ± 0.1	0.7 ± 0.1	0.7 ± 0.1	0.53
Myristic acid (14:0) (g)	2.2 ± 0.1	2.1 ± 0.1	2.2 ± 0.1	2.3 ± 0.1	2.3 ± 0.1	0.12
Palmitic acid (16:0) (g)	14.4 ± 0.2	14.1 ± 0.2	14.7 ± 0.2	15.2 ± 0.3	15.6 ± 0.3 *	<0.001
Stearic acid (18:0) (g)	6.8 ± 0.1	6.7 ± 0.1	7.0 ± 0.1	7.3 ± 0.2	7.5 ± 0.2 *	<0.001
MUFA, total (g)	29.4 ± 0.4	29.4 ± 0.4	30.0 ± 0.4	31.0 ± 0.6	31.8 ± 0.7 *	0.001
Oleic acid (18:1n-9) (g)	27.4± 0.4	27.5 ± 0.4	28.0 ± 0.4	28.8 ± 0.6	29.6 ± 0.7 *	<0.01
PUFA, total (g)	17.2 ± 0.4	18.1 ± 0.4	18.1 ± 0.3	17.9 ± 0.5	18.9 ± 0.6 *	0.03
LA (18:2n-6) (g)	15.2 ± 0.3	16.0 ± 0.3	16.0 ± 0.2	15.9 ± 0.4	16.7 ± 0.5 *	0.03
ALA (18:3n-3) (g)	1.5 ± 0.1	1.6 ± 0.0	1.6 ± 0.0	1.6± 0.0	1.6 ± 0.1	0.20
EPA (20:5n-3 (g)	0.03 ± 0.00	0.03 ± 0.00	0.04 ± 0.0	0.05 ± 0.01	0.03 ± 0.01	0.42
DPA (22:5n-3) (g)	0.02 ± 0.00	0.02 ± 0.00	0.02 ± 0.00	0.02 ± 0.00	0.02 ± 0.00	0.19
DHA (22:6n-3) (g)	0.05 ± 0.00	0.07 ± 0.01 *	0.07 ± 0.01	0.08 ± 0.02	0.06 ± 0.01	0.41
LCn-3, total (g)	0.10 ± 0.01	0.12 ± 0.01	0.13 ± 0.02	0.16 ± 0.03	0.10 ± 0.02	0.36

^1^ Data are presented as mean ± SEM; * *p* < 0.05 compared to the Normal Weight category. SFA, saturated fatty acid; MUFA, monounsaturated fatty acid, PUFA, polyunsaturated fatty acid; LA, linoleic acid; ALA, alpha linolenic acid; EPA, eicosapentaenoic acid; DPA, docosapentaenoic acid; DHA, docosahexaenoic acid; LCn-3, long chain omega-3.

**Table 10:** Dietary intake of energy, macronutrients, and fatty acids by BMI category: United States, NHANES 2005–2012 for adults ≥ 19 years of age with >High School education (*n* = 9772) ^1^.

Nutrient	Normal Weight *n* = 3085	Overweight *n* = 3266	Obese—Grade 1 *n* = 1958	Obese—Grade 2 *n* = 829	Obese—Grade 3 *n* = 634	*p* for Linear Trend
Energy (kcal)	2179 ± 31	2208 ± 20	2155 ± 23	2206 ± 38	2125 ± 41	0.32
Carbohydrate (g)	266 ± 1	258 ± 1 *	254 ± 2 *	257 ± 3 *	258 ± 3	0.02
Protein (g)	82 ± 1	85 ± 1 *	85 ± 1 *	84 ± 1	87 ± 2 *	0.03
Fat, total (g)	79 ± 1	82 ± 1 *	84 ± 1 *	86 ± 1 *	85 ± 1 *	<0.0001
SFA, total (g)	25.7 ± 0.3	26.7 ± 0.2 *	27.2 ± 0.3 *	28.0 ± 0.4 *	28.7 ± 0.5 *	<0.0001
Lauric acid (12:0) (g)	0.8 ± 0.0	0.8 ± 0.0	0.7 ± 0.0	0.9 ± 0.1	0.8 ± 0.0	0.18
Myristic acid (14:0) (g)	2.2 ± 0.0	2.2 ± 0.0	2.2 ± 0.0	2.3 ± 0.1	2.5 ± 0.1 *	<0.001
Palmitic acid (16:0) (g)	13.9 ± 0.2	14.4 ± 0.1 *	14.8 ± 0.2 *	15.1 ± 0.2 *	15.9 ± 0.2 *	<0.0001
Stearic acid (18:0) (g)	6.4 ± 0.1	6.8 ± 0.1 *	7.0 ± 0.1 *	7.2 ± 0.1 *	7.3 ± 0.1 *	<0.0001
MUFA, total (g)	28.8 ± 0.2	29.8 ± 0.2 *	30.7 ± 0.3 *	31.2± 0.4 *	30.7 ± 0.4 *	<0.001
Oleic acid (18:1n-9) (g)	26.9 ± 0.2	27.8 ± 0.2 *	28.7 ± 0.3 *	29.1 ± 0.4 *	28.6 ± 0.4 *	<0.001
PUFA, total (g)	18.2 ± 0.3	18.6 ± 0.2	18.9 ± 0.3	19.3 ± 0.4	18.3 ± 0.4	0.51
LA (18:2n-6) (g)	16.1 ± 0.2	16.4 ± 0.2	16. 8 ± 0.3	17.1 ± 0.4	16.1 ± 0.4	0.50
ALA (18:3n-3) (g)	1.6 ± 0.0	1.7 ± 0.0	1.6 ± 0.0	1.7 ± 0.0	1.6 ± 0.0	0.75
EPA (20:5n-3 (g)	0.05 ± 0.01	0.05 ± 0.00	0.04 ± 0.00 *	0.04 ± 0.01	0.05 ± 0.01	0.44
DPA (22:5n-3) (g)	0.02 ± 0.00	0.02 ± 0.00	0.02 ± 0.00	0.02 ± 0.00	0.02 ± 0.00	0.56
DHA (22:6n-3) (g)	0.09 ± 0.01	0.08 ± 0.01	0.07 ± 0.01	0.08 ± 0.01	0.08 ± 0.01	0.49
LCn-3, total (g)	0.16 ± 0.01	0.15 ± 0.01	0.13 ± 0.0	0.14 ± 0.02	0.15 ± 0.03	0.54

^1^ Data are presented as mean ± SEM; * *p* < 0.05 compared to the Normal Weight category. SFA, saturated fatty acid; MUFA, monounsaturated fatty acid, PUFA, polyunsaturated fatty acid; LA, linoleic acid; ALA, alpha linolenic acid; EPA, eicosapentaenoic acid; DPA, docosapentaenoic acid; DHA, docosahexaenoic acid; LCn-3, long chain omega-3.
